# Radiotherapy treatment modification for prostate cancer patients based on PSMA-PET/CT

**DOI:** 10.1186/s13014-022-01989-5

**Published:** 2022-01-29

**Authors:** Vasileios Karagiannis, Viktor Wichmann, Juha Saarinen, Natalja Eigeliene, Heidi Andersen, Antti Jekunen

**Affiliations:** 1 Department of Oncology, Vaasa Central Hospital, Hietalahdenkatu 2-4, 65130 Vaasa, Finland; 2 Department of Oncology and Radiotherapy, University of Turku, Turku, Finland; 3 Tema Cancer, Karolinska University Hospital, Stockholm, Sweden

**Keywords:** Prostate cancer, PSMA-PET/CT, Decision-making in treatment planning, Radiation therapy

## Abstract

**Background:**

Prostate cancer is the most common cancer among men, and its diagnosis and treatment are improving. Our study evaluated how PSMA-PET/CT prior to treatment planning might improve the optimal management of prostate cancer radiotherapy.

**Methods:**

This retrospective pilot study included 43 prostate cancer (PCa) patients referred to our radiation oncologist department, from the urology department, for radiation therapy. 18F-PSMA-PET/CT was ordered by the radiation oncologists mainly due to the lack of resent image staging. The patients were divided into three different groups according to their initially planned treatments: radical radiation therapy (RT) (newly diagnosed PCa patients), salvage RT (patients with biochemical recurrence after radical prostatectomy), or oligometastatic RT (oligometastatic PCa patients with good response after systemic treatment).

**Results:**

Following PSMA-PET/CT, the initially planned RT was changed for 60.5% of the patients due to new findings (metastases and/or recurrent disease). The final treatment choice was effected by PSMA-PET/CT outcome in 60.5% (26/43) of the patients, and in 50% (16/32) of patients, the radiation treatment plan changed following PSMA-PET/CT. Only 39.5% (17/43) of the patients who underwent PSMA-PET/CT were treated according to their initial treatment plans.

**Conclusions:**

Our results indicate that PSMA-PET/CT impacts treatment decisions and the selection of RT as well as adjuvant treatment protocols in the management of prostate cancer.

## Introduction

Prostate cancer (PCa) is the most common cancer among men, but it can often be treated successfully [1]. In the last decade, tremendous progress in medical science and technology has been made. For PCa, new cancer drugs have been adopted in clinical practice, and patient treatment outcomes have improved. In particular, the diagnosis and management of follow-up are changing because prostate-specific membrane antigen positron emission computed tomography (PSMA-PET/CT) imaging has become available in clinical practice [2]. PSMA-PET/CT is highly effective in finding active lesions. Based on its imaging results, individually tailored radiation therapy (RT) for PCa can be delivered, based on real cancer activity rather than on the indirect measures of the size and growth of malignant lesions, as calculated from computed tomography (CT) scans and magnetic resonance imaging (MRI). PSMA-PET/CT may change our way of performing RT in PCa, as it provides more information on the extent of the cancer, especially in the detection and treatment of lymph node metastases and local recurrences after prior therapies and in controlling oligometastases. However, clinicians are still waiting for conclusive prospective randomized trials with long-term clinical data comparing current practice based on CT and MRI to PSMA-PET/CT guided RT [3].

The advent of PSMA-PET/CT has revolutionized RT for PCa [3]. PSMA-PET/CT has a higher specificity and sensitivity for the detection of tumor lesions than stand-alone CT, MRI and choline-PET. It offers promising opportunities for treatment individualization and can aid clinicians in making the right treatment decisions [4, 5]. Due to the relative novelty of this radiotracer, there is steadily increasing clinical evidence for the implementation of PSMA-PET/CT for clinical decision-making [6–10].

Determining the precise diagnosis before planned treatment is crucial to the delivery of optimal PCa treatment without delays. Usually, patients who first visit an RT oncologist have had blood analyses and PSA levels drawn, and most of them have also had body CT and/or prostate MRI scans. Unfortunately, delays in the diagnostic and decision-making processes may lead to upstaging during the diagnostic process. Fresh scans are needed for RT planning, and disease progression may have taken place.

We aimed to conduct a small pilot study to investigate whether routinely performed PSMA-PET/CT before planned RT could result in changes in decision-making regarding treatment and thus improve outcomes for PCa patients.

## Materials and methods

Patients in this study were treated at the Oncology Clinic of Vaasa Central Hospital, which provides radiotherapy to 6.7% of the Finnish population in the middle of Finland. Our clinic gives RT to approximately 1000 patients annually. In 2019, there were 585 new PCa diagnoses in our area, and 277 patients with PCa were treated in our RT unit [11].

Retrospectively, we focused on PCa patients treated in our RT unit from October 1^st^, 2019, until December 2020. During that time, 361 patients had a first visit for radiation treatment planning. 43 of them (12%) had PSMA-PET/CT, which was ordered by our RT oncologists if they assessed insufficient imaging information or suspected disease progression. The patients were sent to radiation oncology department for radiation treatment planning by the urologists.

Data on patients’ demographics (age) and cancer characteristics (TNM stage, Gleason Score, initial PSA, PSA pre PSMA-PET/CT, ADT) were retrospectively recorded from the hospital charts. Also, PSMA- PET results were manually collected.

All patient undergoing PSMA-PET/CT had tomographic images obtained from the skull vertex to knees following the intravenous injection of about 250 MBq F-18 labelled PSMA-1007 ligand. A low dose CT scan and PET was performed (GE Discovery MI PET/CT) 60 min after injection. Positive findings in PET imaging were constituted if standardized uptake values maximum (SUVmax) was 5 or above and if areas of PSMA activity had anatomically corresponding lesions. Nuclear medical physician interpreted imaging.

The hospital approved the study, according to Finnish legislations for research ethics committee approval was not needed.

## Results

Altogether, 43 PCa patients with PSMA-PET/CT imaging to confirm disease stage were included to the study. Patients could be divided into newly diagnosed PCa 48.83% (n = 21), PCa patients with biochemical recurrence after radical prostatectomy 39.53% (n = 17), and oligometastatic PCa patients responded to systemic treatment 11.9% (n = 5). All the patients were men with histologically verified PCa. They had WHO performance status 0 to1. The Gleason score was 6–7 in 24 patients and 8–10 in 18 patients (Table 1).

**Table 1:** Patient characteristics

Characteristics	All	Radical RT group	Salvage RT group	Oligomts RT group
*Patient groups*				
Patients (n)	43	21	17	5
Age (years), (range)	70 (58–84)	72 (65–81)	69 (58–84)	68 (65–71)
*Gleason score*				
6–7	24	14	8	2
8–9	18	7	8	3
*TNM stage*				
Tx	3	2	1	3
T1-2	25	9	11	2
T3-4	18	10	5	2
N0/x	38	20	16	3
N1-2	5	1	1	3
M0/x	41	21	17	2
M1	2	–	–	
Initial PSA (ng/ml) median (range)	28.4 (6.3–530)	52.3 (11–530)	10.4 (6.3–21)	22.6 (6.9–52)
PSA pre PSMA-PET/CT (ng/ml) median (range)	4.34 (0.1–30.6)	9.4 (0.2–30.6)	2.8 (0.1–21.8)	0.8 (0.1–1.9)
ADT at time of PET	27	21	1	5
PSMA-PET/CT revealed metastases (LN, Bones, FR)	30	11	15	4
Lymph nodes	10	4	7	1
Bones	6	3	1	2
Lymph nodes and bones	5	4	–	1
FR (FR + nodes)	6 (1)	–	6 (1)	–

PSA, prostate-specific antigen; PSMA, PET/CT prostate-specific membrane antigen positron emission tomography/computed tomography; ADT, androgen deprivation therapy; LNs, lymph nodes; FR,fossa recurrence; RT, radiation therapy; Oligomts, oligometastatic; Salvage RT, post-prostatectomy RT

**Fig. 1:**
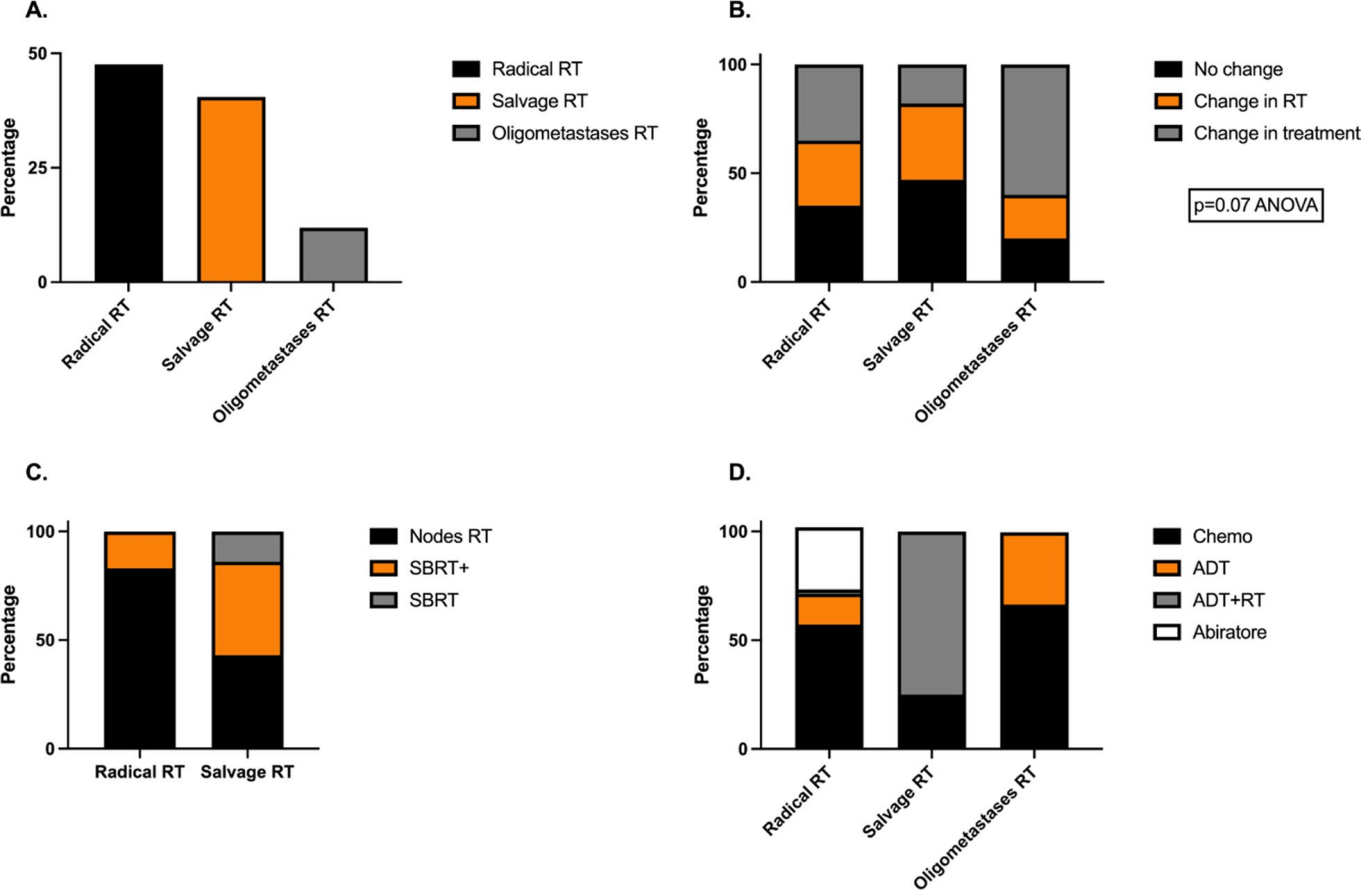
Influence of PSMA-PET/CT results on initially planned treatment decisions for prostate cancer patients. *Abbreviations:* RT—radiotherapy, ChT—chemotherapy, ADT—androgen deprivation therapy, SBRT—stereotactic body radiation. **A** Groups of patients with initially planned treatment by radiation oncologist, as it was requested from the urologist. Overall, 43 PCa patients were divided into three groups according to their primary treatment plans: Radical RT group: 48.83% (n = 21) newly diagnosed PCa patients with initial treatment intention radical/curative RT. Salvage RT group: 39.53% (n = 17) PCa patients with biochemical recurrence after radical prostatectomy and initial treatment intention post prostatectomy RT and Oligometastases RT group: 11.9% (n = 5) oligometastatic PCa patients responded to systemic treatment and initial treatment intention RT to the metastases. Due to the lack of recent/present information on disease status, e.g., CT or MRI scans were performed some time ago or never, radiation oncologists initially made treatment plans; then, to confirm that they made the right decisions, they requested a PSMA-PET/CT for each patient before initiating therapy. **B** Changes in the primary treatment plan after PSMA-PET/CT scan results were observed in every group. In the Radical RT group (n = 21), 33.3% (7/21) proceeded with systemic treatment (ChT, ADT and/or hormonal treatment). 14 pts (14/21) were treated curatively. In 28.6% (6/21) of them, the RT plan was changed and only 38.1% (8/21) went forward as planned. In the Salvage RT group (n = 17), 47% (8/17) of the patients continued with systemic treatment ± RT. 53% (8/15) of the patients treated by RT the RT plan was changed. Only 41% (7/17) of the patients continued as planned. In the Oligometastatic RT group, the majority (60%, 3/5) underwent systemic treatment, and 20% (1/5) and 20% (1/5) of RT plans changed or remained as planned. **C** The patients for whom the RT plan was changed came from radical and salvage RT groups. The radical RT patients for whom the RT plan changed (n = 6), additional lymph node (LN) radiation 83.3% (5/6), and SBRT 16.6% (1/6). In the salvage RT group the RT plan change (n = 8), 62.5% (5/8) additionally received LN radiation 25% (2/8) and SBRT 37,5% (3/8), salvage RT plus boost 25% (2/8) while 12.5% (1/8) received only SBRT. **D** Different systemic treatments were given to patients in every group. Among the patients from the radical RT group for whom systemic therapy was added (n = 7), 57% (4/7) received ChT, 14% (1/7) received ADT, and 28.6% (2/7) received hormonal therapy (abiterone). Among the salvage RT group patients, 25% (2/8) received ChT, and the vast majority 75% (6/8) received ADT + RT. Among the oligometastatic group, 66% (2/3) received ChT, and 34% (1/3) received ADT

The main reasons for the additional imaging were as follows: patients with high risk PCa but not well staged, newly diagnosed PCa patients or patients with biochemical recurrence after radical prostatectomy (no images had been performed within 6 months), PSA had aggressively increased without clear reason, and patients became symptomatic (Table 1). These patients were grouped into three separate groups according to their initially treatments' request from the urologists group A—patients for radical RT (n = 21). Patients send to radiation department, for initial radical/curative RT group B—patients for salvage RT (n = 17). Patients with biochemical recurrence after radical prostatectomy send for post prostatectomy/salvage RT or group C-RT for oligometastatic disease (n = 5) Patients with oligometastatic PCa, with good response after systemic treatment (Fig. 1A). Oligometastatic disease was defined as less than 5 metastases in less than three organs.

According to the results, the final treatments changed substantially in all the groups. In group A, 33.3% (7/21) of patients were diagnosed with multiple metastatic lesions and started systemic treatment with docetaxel or hormonal therapy with androgen deprivation therapy (ADT). In 28.6% (6/21) of patients, the RT treatment plan was changed (Fig. 1B).

In group B, 47% (8/17) of the patients continued with systemic treatment ± RT. For 53% (8/15) of the patients which finally treated by RT, the RT plan was changed (Fig. 1B).

Last, in group C, 60% of the patients (3/5) underwent systemic therapy; 2/3 started chemotherapy, and 1/3 received ADT. In 20% (1/5) of patients, non-metastatic disease was documented by PSMA-PET/CT imaging and the patients received curative RT treatment (20 × 3 Gy prostate RT), one oligometastatic patient was treated by hypofractionated prostate RT 60/3 Gy and bone metastasis 50/2.5 Gy and SBRT 3X9 Gy bone metastasis. (Fig. 1B, D).

In group A seven patients were initially set to receive radical/curative RT but treatment was later changed to systemic therapy, which stands out as this comprised the proportion of high-risk patients. (Table 2), During the first visit to the RT oncologist, the tumor sizes were cT3 (n = 4) and cT4 (n = 3), and the Gleason scores were Gleason 7 (n = 4) and Gleason 8 (n = 3). For the initial staging pelvic MRI (n = 2), SPET TT (n = 1), bone Scan (n = 5) and body CT (n = 2) were performed. Two patients were suspicious having bone metastasis according to the bone scan and the body CT. However, all patients had neoadjuvant ADT, and PSA decreased (after neoadjuvant ADT) compared to previous PSA levels. All the patients were sent by the urologists for curative local treatment. RT oncologist ordered PSMA-PET/CT due to the high probability of metastases (high risk patients). In addition, PSA value increased (n = 2), the suspicious findings in the initial studies (n = 2), enlarged pelvic nodes in planning CT (n = 1) and not good response to the neoadjuvant ADT (n = 2). PSMA-PET/CT revealed lymph nodes metastases (n = 1), in bones (n = 2), and bones metastases and lymph nodes metastases (LNs) (n = 4).

**Table 2:** Radical RT patient group characteristics for whom RT was preplaced by systemic therapy

Characteristics	Radical RT group
Patients (n)	7
*Gleason score*	
6–7	4
8–9	3
*TNM stage*	
cT3	4
cT4	3
Initial PSA (ng/ml) (median)	106.5 (12.5–530)
Patients who received neo-adjuvant ADT (n)	7
Post-ADT PSA (ng/ml) (median)	3.0 (0.2–7.5)
Pre-PSMA-PET/CT PSA (ng/ml) (median)	8.7 (0.2–30)
*PSMA-PET/CT revealed metastases in*	
Lymph nodes	1
Bones	2
Lymph nodes and bones	4

Pts, patients; PSA, prostate-specific antigen; PSMA-PET/CT, prostate-specific membrane antigen positron emission tomography/computed tomography; ADT, androgen deprivation therapy; LN, lymph nodes; RT, radiation therapy

57% (4/7) of the patients received chemotherapy, 14.2% (1/7) received ADT, and 28.6% (2/7) received abiraterone (Fig. 1D).

Finally, 66.6% (14/21) of the patients, from group A, were treated by RT. The RT plan was adapted to PET findings. Hypo fractionated radical prostate RT 60/3 Gy and SBRT to the bone metastases 3 × 9 Gy (n = 1), Hypo fractionated RT; prostate 60/3 Gy, 56/2.8 Gy to PET + pelvic nodes and pelvic RT 46/2.3 Gy (n = 1). Hypofractionated radical prostate RT 60/3 Gy (n = 3), Hypo fractionated RT; prostate 60/3 Gy, seminal vesicular 48/2.4 Gy and pelvic RT 46/2.3 Gy (n = 9) (n = 1 seminal vesicular invasion, n = 4 suspicious for pelvic nodes invasion).

In group B according to the radical prostatectomy PAD report pT1-2 (n = 11), pT3-4 (n = 5), pTx (n = 1), pN1 (n = 1), pN0/Nx (n = 9) and Gleason scores 6–7 (n = 8), 8–9 (n = 8). Positive surgical margins in 5 patients. Based on PSA values elevation, those patients who were sent to receive salvage RT for biochemical recurrence after radical prostatectomy, without any additional images. The interval between primary treatment and reference for salvage RT was between 6 months and 3 years (n = 1 more than 15 years and n = 1 more than 10 years). Median postoperative PSA 0.72 (ng/ml) and median PSA, just before PSMA-PET/CT, 2.83 (ng/ml).

PSMA-PET/CT revealed metastases or local recurrence in 15/17 patients. Local recurrence (n = 6), LNS metastases (n = 7), bone metastases (n = 1), and local recurrence and LN metastases (n = 1). The median PSA levels before PSMA-PET/CT in PET-positive patients were 1.62, and in PET-negative patients 0.55 ng/ml (Table 3).

**Table 3:** Salvage RT patient group characteristics

Characteristics	Salvage RT group
Patients (n)	17
Age (median)	69 (58–84)
Radical prostatectomy	17
*TNM stage*	
pT1-T2	11
pT3a-b	5
pT4	1
pN1	1
pNx	9
Mx	17
*Gleason score*	
6–7	8
8–9	8
Positive surgical margins after radical prostatectomy	5
Pre-PSMA-PET/CT PSA (ng/ml) (median)	2.8 (0.1–21.8)
Postoperative PSA (ng/ml) (median)	0.7 (0–7)
PSA (ng/ml) at PSMA-PET/CT in PET positive pts	1.6 (0.4–13.8)
PSA (ng/ml) at PSMA-PET/CT in PET negative pts	0.6 (0.1–1)
*PSMA-PET/CT revealed metastases in*	
FR only	6
Lymph nodes	7
Bones	1
FR and lymph nodes	1

Pts, patients; PSA, prostate-specific antigen; PSMA-PET/CT, prostate-specific membrane antigen positron emission tomography/computed tomography; ADT, androgen deprivation therapy; LN, lymph nodes; FR, fossa recurrence; RT, radiation therapy

The RT plan was modified according to PSMA-PET/CT findings. Late salvage RT for local recurrence (n = 2) (70/2 Gy to the prostate bed and additional SIB 73.5/2.1 Gy), late salvage RT 66–70/2 Gy plus pelvic nodes radiation 54 Gy and 50.4 Gy (n = 2). Late salvage RT (66-70 Gy) plus SBRT 5 × 6 Gy to the PET + nodes (n = 3), late salvage RT 70 Gy (n = 6), pelvic nodes radiation 46/2.3 Gy and SIB to the PET + pelvic nodes 60/3 Gy (n = 1) and SBRT to PET + nodes 5 × 6 Gy (n = 1).

11.7% (2/17) of the patients received chemotherapy instant of salvage RT, and 35% (6/17) received ADT + RT (Fig. 1D).

## Discussion

Our study is the first real world report in Finland to show how important it is to know the results of the PSMA-PET/CT before planning RT. In Finnish guidelines, until now, PSMA-PET/CT has no indication in initial staging for PCa patients and still it is not used regularly for investigation of biochemical recurrences after radical prostatectomy/radiotherapy. According to our results, the treatment plan changed for more than half of the patients due to new findings on the PSMA-PET/CT. Most of these patients had high-risk PCa. Of those treated with RT, PSMA-PET/CT changed the radiation plan and impacted treatment decisions.

In accord with our results, several studies have already shown that the implementation of PET/CT resulted in a significant management change rate in the postoperative setting, ranging from 35 to 64% [12, 13]. Under-staging, especially in men with high-risk PCa and recurrent PCa after radical prostatectomy or radical radiation, leaves metastases untreated and may lead to poor treatment outcomes. Personalized treatment decisions must be based on evidence and not only on mathematical models and risk factors. *Guevelou *et al*.* reviewed the potential impact of restaging based on PSMA-PET/CT changes in the management of recurrent prostate cancer after radical prostatectomy (RP) [12]. PSMA-PET/CT has proven its accuracy in restaging patients in either the local, nodal, or metastatic setting [12]. Previously, RT treatment schedules for PCa have been established by relying on randomized trials using statistical risk calculations, nomograms, and conventional imaging to characterize patients and guide treatment decisions. Definitive radiation therapy (dRT) is an effective initial treatment for intermediate-risk (IR) and high-risk (HR) PCa. PSMA-PET/CT is superior to standard-of-care imaging, such as CT, MRI, and bone scans, for detecting regional and distant metastatic PCa. PSMA-PET/CT, thus has the potential to guide patient selection and planning for RT and to improve patient outcomes [14].

The expedited systemic review published by *Petersen *et al. showed the promising diagnostic accuracy of PSMA-PET/CT for primary lymph node staging with pathology as a reference in intermediate- and high-risk PCa. The sensitivity ranged from 23 to 100%, the specificity ranged from 67 to 100%, and the positive predictive value ranged from 41 to 100% [15]. A systematic review by *Koschel S *et al. was based mainly on retrospective studies suggesting that Ga-PSMA-PET/CT was superior to bone scans in identifying bone metastases [16]. The Australian prospective, multicenter, randomized prePSMA study showed the diagnostic superiority of PSMA-PET/CT compared with body CT and bone scan with SPECT/CT, enabling the comparison of regional and distant metastases when staging men with high-risk localized PCa. A total of 302 patients from 10 centers were randomized into two groups: the conventional imaging group (pelvis CT, bone scan, and SPECT/CT) and the PSMA-PET/CT group. PSMA-PET/CT had greater accuracy than conventional imaging (92% vs. 65%; *p* < 0.0001. Conventional imaging had a lower sensitivity (38% vs. 85%) and specificity (91% vs. 98%) than PSMA-PET/CT. Thirty percent of the men studied had metastases in pelvic nodes or elsewhere [17].

Based on the evidence above, it is important to have accurate image staging in PCa patients before starting RT to detect oligometastatic disease. HORRAD, STAMPEDE and CHAARTED [18–20] also suggested that the addition of prostate-targeted RT (PTT-RT) to standard ADT might be beneficial in patients with a low metastatic burden. Patients with fewer than five bone lesions showed improvements in survival. Accumulating evidence has indicated that fractionated radiotherapy can result in distant nonirradiated, abscopal, and tumor regression. This abscopal effect may be one of the potential reasons why PTT-RT is beneficial in patients with a metastatic burden. Additionally, in ORIOLE, improvement in the median PFS (*P* = 0.002) was detected in the stereotactic ablative radiation group vs. the observation group in men with 3 or fewer extracranial metastases [21]. Last, the SABR session was feasible and associated with low morbidity in POPSTAR (single fraction of 20-Gy SABR to each lesion to men treated for oligometastatic PCa). The 2-year LPFS was 93%, and the 2-years DPFS was 58% [22].

Oligoprogressive disease represents the growth of a few lesions in a background of otherwise controlled metastatic disease. For example, *Pasqualetti *et al. reported a prospective observational study assessing the role of image-guided stereotactic radiotherapy (IG-SBRT) [23]. The estimated 12- and 24-month local control ratios were 98.7% and 97.4%, respectively. In the STOMP trial, 62 patients with asymptomatic PCa and 3 or fewer extracranial metastatic lesions were randomized (1:1) to either surveillance or metastasis-directed therapy (MDT) of all detected lesions SBRT (stereotactic body radiotherapy). The primary endpoint was androgen deprivation therapy (ADT)-free survival. The median ADT-free survival was 13 months for the surveillance group vs. 21 months for the MDT group (*P* = 0.11). No grade 2 to 5 toxicity was reported [22]. In another prospective clinical trial, 199 PCa patients (with up to 5 metastases) were enrolled to receive fractionated SBRT (50 Gy in 10 fractions) for each visible lesion. A prostate-specific antigen (PSA) decline was induced in 75% of patients. The proportion of patients not requiring treatment escalation 2 years following SBRT was 51.7% [24]**.**

According *Mazolla *et al. the preliminary data of MRI guided SBRT, RT was safe and well tolerated and no acute G2 or higher toxicities were observed. 20 castration sensitive oligorecurrent PCa patients (lymph nodes and pelvic bones) underwent MR-guided SBRT (1.5 T MRI-Linac), mean total dose of 35 Gy (range, 25–36.25 Gy) in 5 fractions. Median pre-SBRT PSA value 1.16 ng/mL (range 0.27–8.9 ng/ml). During the first follow up, detectable PSA was found in 16 out of 20 patients. In post treatment PSMA PET/CT PR in 6 cases and CR in 10 cases [25].

The use of MDT in oligoprogressive disease represents an area of recent interest considering the evolving and improving systemic therapies such as targeted agents and immunotherapies that can often lead to long periods of disease control. The goal of MDT in these instances is to sterilize resistant cancer cell clones. Many ongoing trials have investigated the role of MDT in oligoprogressive disease: SOC systemic therapy + / − RT in CRPC [26], SABR + ADT [27], SABR + abiraterone [28], SABR + ipilimumab [29], and SABR + durvalumab [30].

However, again, there is a need for clinical studies challenging the use of RT in treatment schemes and comparing the efficacy of early systemic therapy to that of alternatives. In the SPPORT trial;1,792 men with persistently detectable or rising PSA levels and evidence of positive surgical margins after prostatectomy in the prostate bed, pelvic lymph nodes, or elsewhere in the body after prostatectomy were randomized into 3 groups: prostate bed radiation therapy (PBRT) alone, PBRT plus short-term ADT (STAD), and PBRT plus pelvic lymph node radiotherapy (PLNRT) plus ADT [31]. After 8 years of follow-up, distant metastases were found in 45 patients from the PBRT-only arm, in 38 patients from the PBRT + ADT arm, and in 25 patients from the PLNRT + PBRT + ADT arm.

PSMA-SRT is the first ongoing randomized prospective phase III trial [32]. The purpose of this study was to evaluate the success rate of SRT in preventing the recurrence of PCa after prostatectomy with and without planning based on PSMA-PET/CT. A total of 193 patients will be randomized (1:1.3) to standard SRT (control arm 1, n = 90) or will undergo a PSMA-PET/CT scan prior to SRT planning (investigational arm 2, n = 103). The primary endpoint is the success rate of SRT measured as biochemical progression-free survival (BPFS) after initiation of SRT. Biochemical progression was defined by PSA ≥ 0.2 ng/mL and rising. The choice of treating the prostate bed alone vs. the prostate bed and pelvic lymph nodes, with or without ADT, will be selected by the treating radiation oncologist. Patients will be followed until one of the following conditions occurs: biochemical progression, diagnosis of metastatic disease, initiation of any additional salvage therapy, death, or 5 years pass since the date of initiation of randomization. This is the first randomized phase 3 prospective trial designed to determine whether PSMA-PET/CT molecular imaging can improve outcomes in patients with PCa following radical prostatectomy [32].

The strength of our study is its similarity to clinical everyday practice, as it shows how PSMA-PET/CT changes the treatment of patients with PCa when imaging is performed before RT and when the findings are implemented in treatment. In fact, our study is the first report of the impact of findings from PSMA-PET/CT in mostly high-risk PCa patients. Our study limitations are the small number of patients and the lack of a prospective study design or a control arm. In addition, PSMA-positive lesions were not confirmed by biopsy because it was not practical to biopsy numerous bone lesions and not easily adaptable to clinical practice. Similarly, lymph node biopsies were not performed.

Clinical trials with PCa have included limited radiological staging and have particularly not included PSMA-PET/CT. For example, in the CHAARTED study, the investigators did not use PSMA-PET/CT but instead used conventional CT and technetium bone scan imaging and revealed that even crude imaging identified patients with high volume disease who benefited from early docetaxel [18–20]. The lack of data makes it difficult to include imaging recommendations in guidelines. However, some patients with higher stages of disease may have been treated based on the suboptimal assumption of spreading of cancer. Our study also included patients with more extensive disease, as those patients may benefit more from RT. Many studies thus far have shown promising results if treatment is adapted to imaging findings, and many retrospective studies suggest that metastasis-directed therapy (MDT) for oligometastatic PCa improves PFS. Ost et al*.* showed that MDT for patients with oligorecurrent PCa is safe and improves ADT-free survival compared with surveillance [33].

It sounds logical to have systemic therapy initiated at the point when metastases are detected and not to wait for symptoms and lesions to become detectable in ordinary imaging. PEACE V is a randomized controlled phase II trial that attempts to investigate the impact of ADT addition to MDT and to WPRT in pelvic nodal recurrence disease. Patients diagnosed with PET-detected pelvic nodal oligorecurrence (≤ 5 nodes) following radical local treatment for PCa will be randomized in a 1:1 ratio between arm A: MDT and 6 months of ADT or arm B: WPRT added to MDT and 6 months of ADT. The primary endpoint is MFS, and the secondary endpoints include clinical and biochemical progression-free survival (PFS), PCa-specific survival, quality of life (QoL), toxicity, and time to castration-resistant prostate cancer (CRPC) and palliative ADT [34].

Currently, PSMA-PET/CT is being incorporated into the routine care of men with PCa. Understanding its strengths, its limitations, and when it is valuable in guiding clinical care are important to the provision of the best patient care [35]. The place of PSMA-PET/CT should be under academic discussion based on available data, and it should be considered a new gold standard of imaging based on data from retrospective studies. However, the Finnish prospective, nonrandomized single-center trial (PROSTAGE) will try to compare the diagnostic accuracy of PSMA-PET/CT to whole body MRI, SPECT/CT, whole body CT, and bone scan in high-risk PCa for the detection of metastases [36]. The PRIMARY (prospective, multicenter, cross-sectional study) will measure and compare the sensitivity, specificity, positive predictive value and negative predictive value of both multiparametric magnetic resonance imaging (mpMRI) and pelvic-only PSMA-PET/CT vs. targeted prostate biopsy. Additionally, PRIMARY will investigate whether limited (pelvic-only) PSMA-PET/CT in combination with routine mpMRI can reliably discriminate men with clinically significant prostate cancer (csPCa) from those without csPCa [37].

Our results and current literature indicate that PSMA-PET/CT impacts treatment decisions and the selection of RT as well as adjuvant treatment protocols in the management of prostate cancer. PSMA-PET/CT should be adapted from international guidelines to national guidelines.

The incorporation of PSMA-PET/CT imaging in planning will change RT treatment and may improve its likelihood of success. Further prospective randomized studies are urgently needed to confirm that early treatment based on imaging-detected metastases will improve PCa patient outcomes.

## Data Availability

All data generated and analyzed of this study are included in this article.

## Abbreviations

PCa: Prostate cancer
csPCa: Clinically significant prostate cancer
PSMA-PET/CT: Positron emission computed tomography
SUVmax: Standardized uptake values maximum
MRI: Magnetic resonance imaging
mpMRI: Multiparametric magnetic resonance imaging
RT: Radiotherapy
PBRT: Prostate bed radiation therapy
SBRT: Stereotactic body radiation
SABR: Stereotactic ablative radiotherapy
IG-SBRT: Image- guided stereotactic radiotherapy
STAD: Plus short-term ADT
PLNRT: Pelvic lymph node radiotherapy
dRT: Definitive radiation therapy
IR: Intermediate-risk
HR: High- risk
ChT: Chemotherapy
ADT: Androgen deprivation therapy
MDT: Metastasis-directed therapy
